# Internal Microscopic Diagnosis of Accelerated Aging of Proton Exchange Membrane Water Electrolysis Cell Stack

**DOI:** 10.3390/mi11121078

**Published:** 2020-12-04

**Authors:** Chi-Yuan Lee, Chia-Hung Chen, Guo-Bin Jung, Shih-Chun Li, Yi-Zhen Zeng

**Affiliations:** 1 Yuan Ze Fuel Cell Center, Department of Mechanical Engineering, Yuan Ze University, Taoyuan 32003, Taiwan; guobin@saturn.yzu.edu.tw (G.-B.J.); st89309cm@gmail.com (S.-C.L.); a0988276238@gmail.com (Y.-Z.Z.); 2 HOMYTECH Global CO., LTD., Taoyuan 32003, Taiwan; chenjahon@gmail.com

**Keywords:** PEM water electrolysis cell stack, accelerated aging, flexible integrated microsensor, microscopic diagnosis

## Abstract

The hydrogen production reaction of the proton exchange membrane (PEM) water electrolysis cell stack is the reverse reaction of the fuel cell, but the water electrolysis operation requires high pressure, and the high pressure decomposes hydrogen molecules, thus aging or causing failure in the water electrolysis cell stack. In addition, there are five important physical parameters (current, voltage, flow, pressure and temperature) inside the water electrolysis cell stack, which can change the performance and shorten the life of the cell stack. However, the present techniques obtain data only by external simulation or single measurement; they cannot collect the internal real data in operation instantly and accurately. This study discusses the causes for aging or failure, and develops an internal real-time microscopic diagnosis tool for accelerated aging of the PEM water electrolysis cell stack. A flexible integrated (current, voltage, flow, pressure and temperature) microsensor applicable to the inside (high voltage and electrochemical environment) of the PEM water electrolysis cell stack is developed by using micro-electro-mechanical systems (MEMS) technology; it is embedded in the PEM water electrolysis cell stack for microscopic diagnosis of accelerated aging, and 100-h durability and reliability tests are performed. The distribution of important physical parameters inside the PEM water electrolysis cell stack can be measured instantly and accurately, so as to adjust it to the optimal operating conditions, and the local aging and failure problems are discussed.

## 1. Introduction

With the progress of human civilization, as energy becomes more and more important, the demand increases greatly, and people use fossil fuels, such as petroleum, natural gas and coal, to meet the huge demand, so that a lot of CO_2_ is emitted, which induces global warming. Therefore, renewable energy has become the key point of current research, including hydrogen energy, solar energy, hydraulic power, wind power, geothermal, tide and biomass energy. Hydrogen is one of the optimal sustainable energy carriers, and hydrogen is a zero carbon energy carrier; it is applicable to low-carbon transportation and industrial decarbonization. Generally, burning fossil fuels to produce hydrogen will generate a lot of CO_2_, so carbon-free hydrogen production is the present research objective. Electrolytic technology can transform low-carbon electricity, which is unlikely to be stored and transmitted into hydrogen. It is friendlier to the environment than traditional hydrogen production methods (e.g., coal gasification and natural gas reforming). In addition, the intermittent power problems in wind energy and solar energy can be solved, and hydrogen is generated continuously, so as to relieve the outage problem. Renewable energy and electrolytic technology have major significance for promoting the high quality development of the hydrogen energy industry, promoting mass renewable energy consumption and speeding up energy structure transformation [1]. At present, the main hydrogen production methods are the water electrolysis method, the water photo-electrolysis method, fossil fuel hydrogen production, and the industrial surplus hydrogen and biological methods. In comparison to prior water electrolysis, the proton exchange membrane (PEM) water electrolysis cell stack has many advantages, such as less corrosivity, it can be operated at lower voltage, higher current density and higher temperature and pressure, so as to increase efficiency (80–90%). Its defects include high material cost and cross infiltration, and dehumidification is required as the pressure increases and the water vapor occurs. Developing new materials can reduce the total cost of the water electrolyzer to implement rapid commercialization, but it is necessary to understand the internal performance state [2]. In long-term operation of the PEM water electrolysis cell, its internal local physical quantities are distributed unevenly, its performance is influenced, and the membrane material durability is influenced, leading to catalyst corrosion [3,4,5,6,7]. The polymer electrolyte membrane (PEM) process is perceived as a key process for transforming zero-carbon electricity sources into the supply of zero-carbon hydrogen and oxygen for miscellaneous end-uses [3]. The characteristic of PEM electrolysis is very suitable for water splitting using an intermittent power supply [4]. In principle, PEM technology offers the possibility of operating at such high pressures because low permeability membranes can be used as solid polymer electrolytes [5]. The PEM electrolyzer works under low DC voltage, so it can be directly coupled to photovoltaic panels [6]. The PEM electrolysis machine has several advantages compared with alkaline electrolysis machines: much higher current density; high gas purity; improved safety level (no corrosive electrolyte loop); and the possibility of combining fuel cell and electrolysis machine modes [7]. If the input voltage of the PEM water electrolysis cell is too high, the resistance is likely to be too high, and the nonuniform current density causes the hot spot to generate waste heat, so that the system temperature rises, the decomposition of hydrogen molecules is accelerated, the hydrogen concentration drops, the runner plate is damaged, and the aging and performance decay of water electrolysis cell are accelerated. If the flow is too low, the hydrogen production efficiency of PEM water electrolysis cell will decrease, and the heat cannot be transferred outwards. If the voltage is too high, the catalyst will be damaged due to electrochemical corrosion. If the voltage is too low, the reaction will be incomplete [8,9,10,11,12,13,14,15,16,17,18]. The perfluorinated membrane material used in the PEM water electrolysis has high acidity, so expensive noble metals or their oxides are required (platinum for hydrogen evolution reaction or HER for the cathode and Iridium or oxygen evolution reaction (OER) for the anode [8]. On the other hand, operating at a current density that is usually an order of magnitude larger than alkaline electrolysis (at the same efficiency level) can provide a way to reduce capital costs. The effect of flow rate on performance depends to a large extent on the operating temperature in the two flow channel modes [9]. The effect of flow rate on performance depends to a large extent on the operating temperature in the two flow channel modes. At high current densities, the PEM water electrolyzer will form a large amount of gas, which poses a challenge to remove the product and ensure that the electrode receives sufficient water. [10]. Hydrogen cross-over through a membrane electrolyte is a critical safety issue in proton exchange membrane (PEM) electrolysis. Permeated H2 tends to be consumed by oxidation or recombination at the anode [11]. The proton exchange membrane is the weakest part of the PEM electrolyzer regarding long-term performance. A significant thinning of the film in the stack was detected. The dissolution process starts at the interface between the cathode and the membrane, and is most likely triggered and/or enhanced by local stress on the membrane [12]. Most performance losses and most accidents that occur during PEM water electrolysis are usually caused by membrane electrode assembly (MEA) [13]. However, one of the key points is related to system cost and life expectancy. On the one hand, the use of acidic electrolytes and the high working potential of anode and temperature, and on the other hand, material optimization, especially film thickness, have now reached a critical point, requiring a lot of effort to make small improvements [14,15,16,17,18]. The current, voltage, temperature and flow inside the PEM water electrolysis cell will influence its performance [19]. However, the present techniques obtain data only by external simulation or single measurement; they cannot collect the internal real data in operation instantly and accurately. This study uses MEMS technology to further develop a flexible integrated (current, voltage, flow, pressure and temperature) microsensor applicable to the inside of the PEM water electrolysis cell stack, which is embedded in the PEM water electrolysis cell stack for microscopic diagnosis of accelerated aging.

## 2. Research Methods

**Figure 1:**
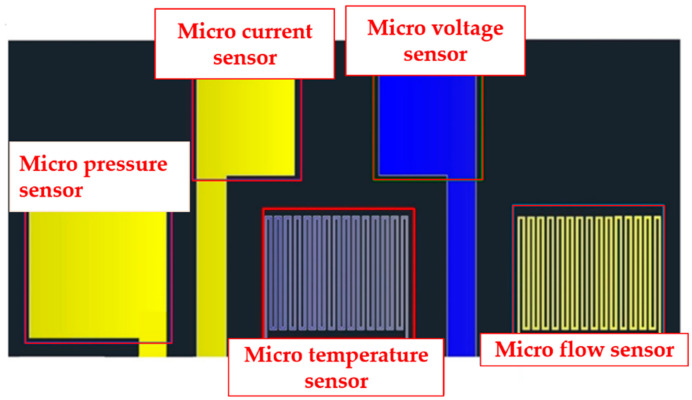
Integrated design drawing of 5-in-1 microsensor.

The micro current, voltage, flow, pressure and temperature sensors are integrated into a 5-in-1 microsensor, as shown in Figure 1, by using MEMS technology, which is embedded in the PEM water electrolysis cell stack; the local distributions of internal current, voltage, flow, pressure and temperature are measured simultaneously, and a 100-h accelerated aging test is performed. Temperature sensing area is 750 μm × 600 μm; voltage sensing area is 600 μm × 600 μm; current sensing area is 600 μm × 600 μm; flow sensing area is 750 μm × 600 μm and pressure sensing area is 850 μm × 850 μm.

### 2.1. Sensing Principle of Micro Temperature Sensor

The sensing principle of micro temperature sensor is that when the ambient temperature rises, as Au has a Positive Temperature Coefficient (PTC), the resistivity increases with temperature; this characteristic results from the “temperature coefficient of resistance” (TCR) of the conductor.

### 2.2. Sensing Principle of Micro Humidity Sensor

The electrode form of the capacitive micro humidity sensor used in this study is the interdigitated electrode structure. There is a humidity sensitive thin film above the electrode. When the water vapor absorbed by the humidity sensitive thin film increases, the dielectric constant increases with ambient humidity; the humidity can be obtained by calculating the changed capacitance value.

### 2.3. Process Development of Flexible Integrated Microsensor

**Figure 2:**
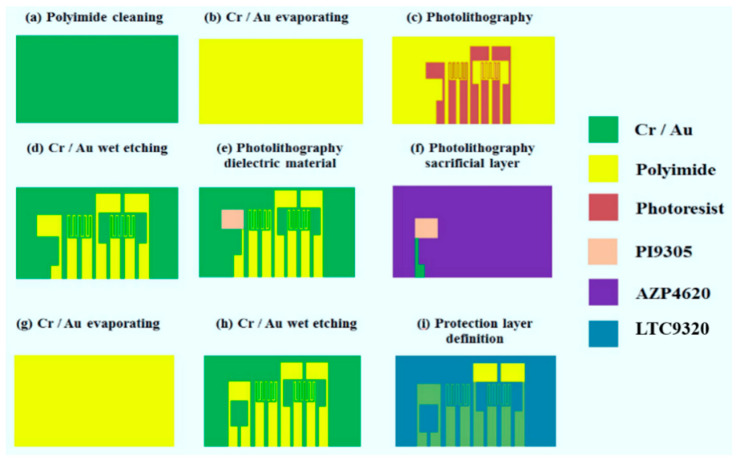
Process chart of flexible integrated microsensor.

The current, voltage, flow, pressure and temperature sensing structures are integrated by using MEMS technology in this study. The process of the flexible integrated microsensor equipment is shown in Figure 2.

(a) PI thin film cleaning

The PI (Polyimide) thin film substrate is cleaned with organic solvent ethanol, and then it is put in the hot organic solvent acetone, and the acetone is volatilized.

(b) Evaporate metal

**Figure 3:**
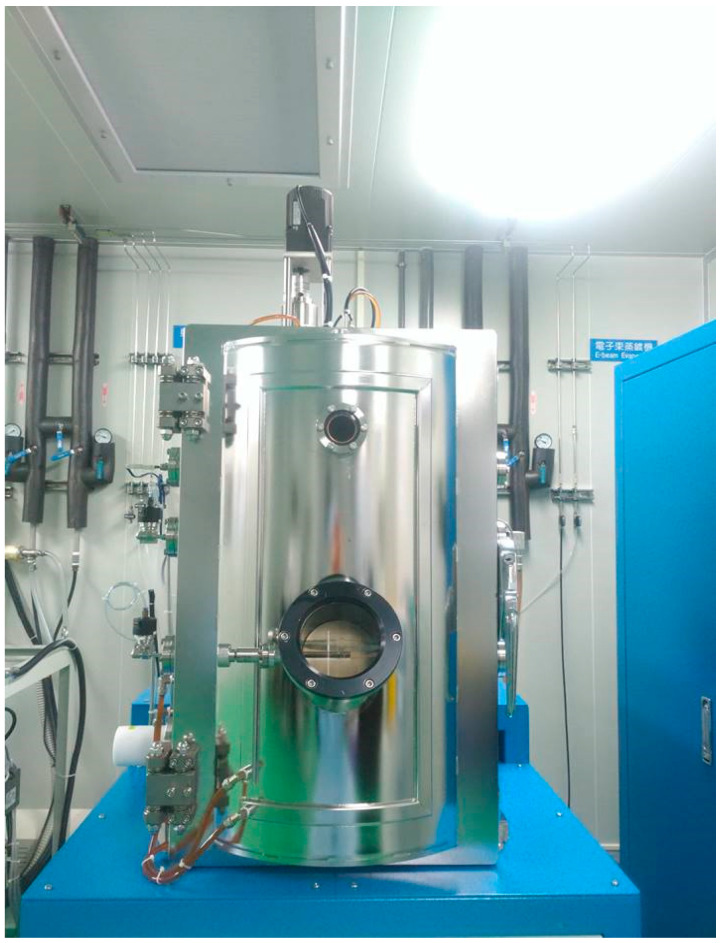
Electron beam evaporator, EBS-500, Junsun technologies Co., Taipei, Taiwan.

The metal is evaporated by electronic beam evaporator as shown in Figure 3; the 100 Å thick Cr and 1000 Å Au are deposited at deposition rate of 0.1 Å/s.

(c) Photolithography

**Figure 4:**
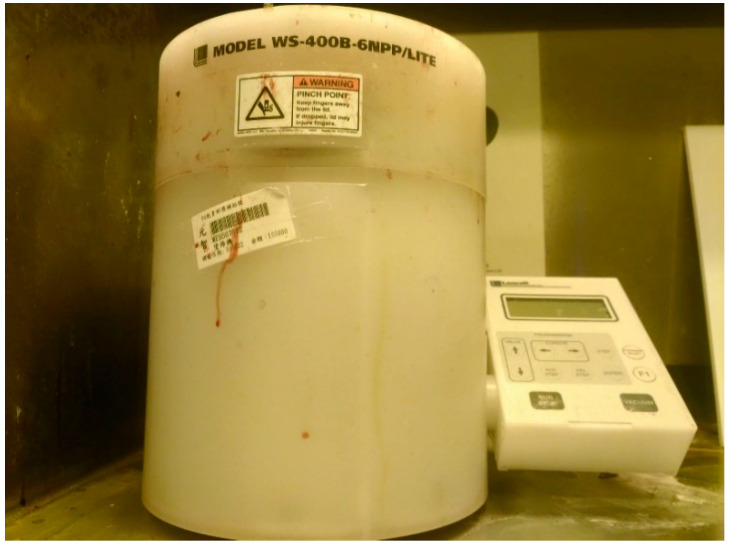
Spin coater.

**Figure 5:**
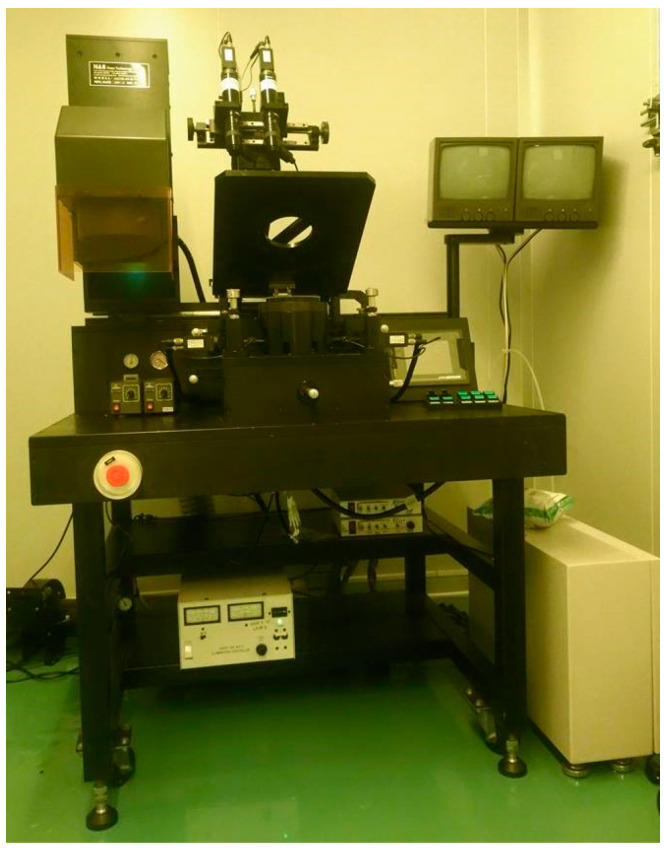
Mask aligner and exposure system, AG-200-4N-D-SM, M&R Nano Technology Co.

The positive photoresist is coated on the sample uniformly by spin coater, as shown in Figure 4, and the pattern of integrated microsensor is defined by using photolithography, as shown in Figure 5.

(d) Metal etching

The photoresistor (AZ^®^ P4620, Merck Co., Darmstadt, Germany) can form a wet etching mask after the photolithography process; the unwanted Au/Cr is corroded by Type-TFA Au etching solution which is manufactured by Transene Electronic Chemicals (Danvers, MA, USA), and by Cr-7T Cr etching solutions, manufactured by OM Group Incorporated (Cleveland, OH, USA), and then the photoresist is removed by acetone.

(e)~(i) Dielectric layer definition, evaporate metal, metal etching and protection layer definition

**Figure 6:**
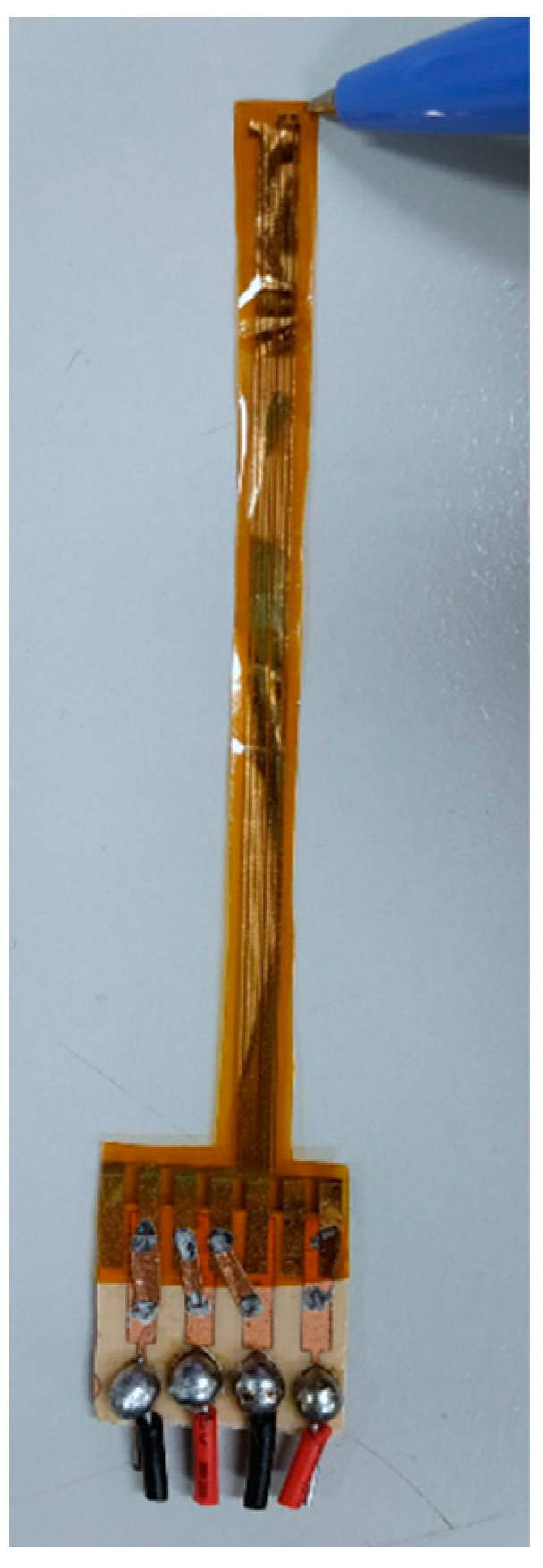
Stereogram of flexible integrated microsensor.

The pattern of the dielectric layer is defined through the aforesaid photolithography process, the Cr/Au metal is evaporated again, and the unwanted metal is removed by wet etching process. Finally, the LTC9320 (FUJIFILM Electronic Materials Co, Hsin-Chu, Taiwan) with high mechanical strength and fit for high chemical environment is used as a protection layer, so as to avoid the flexible integrated microsensor being destroyed by the closing pressure of end plate inside the PEM water electrolysis cell stack. Afterwards, the sensing area of micro voltage and current sensors is defined by using the photolithography process. The stereogram of the flexible integrated microsensor is shown in Figure 6.

### 2.4. Correction of Flexible Integrated Microsensor

**Figure 7:**
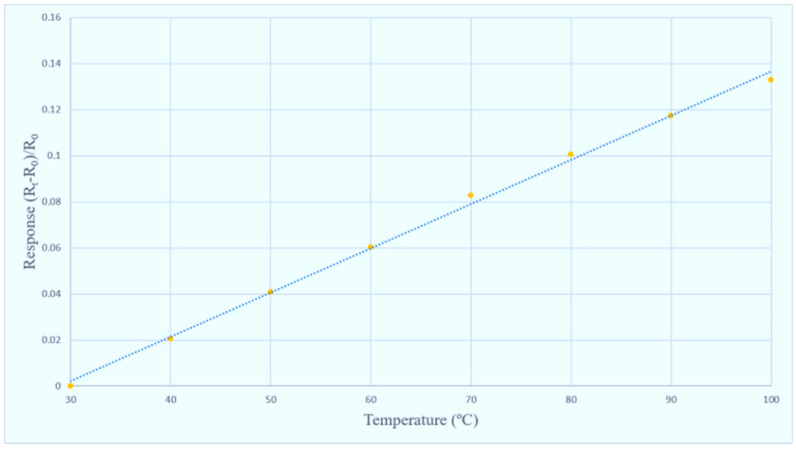
Correction curve of micro temperature sensor.

**Figure 8:**
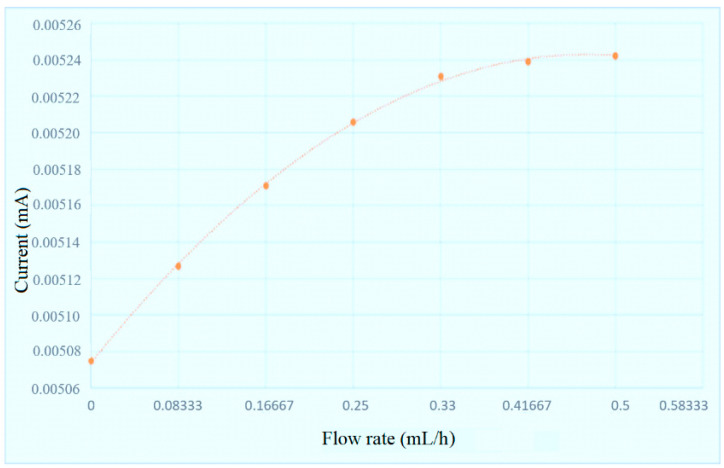
Correction curve of micro flow sensor.

After the sensing structure is successfully integrated into the PI film, the temperature and flow of flexible integrated microsensor are corrected, and the reliability is validated. The flexible integrated microsensor is connected to NI PXI 2575 data capture equipment of National Instruments (NI, Austin, TX, USA) to capture the microsensor data instantly, as well as the resistance and current of micro temperature and flow sensors. The system is measured and controlled by LabVIEW system design software, and the signals are processed and analyzed and exported to the computer. The correction curves of temperature and flow are drawn, respectively, as shown in Figure 7 and Figure 8.

## 3. Accelerated Aging Test for PEM Water Electrolysis Cell Stack 

**Figure 9:**
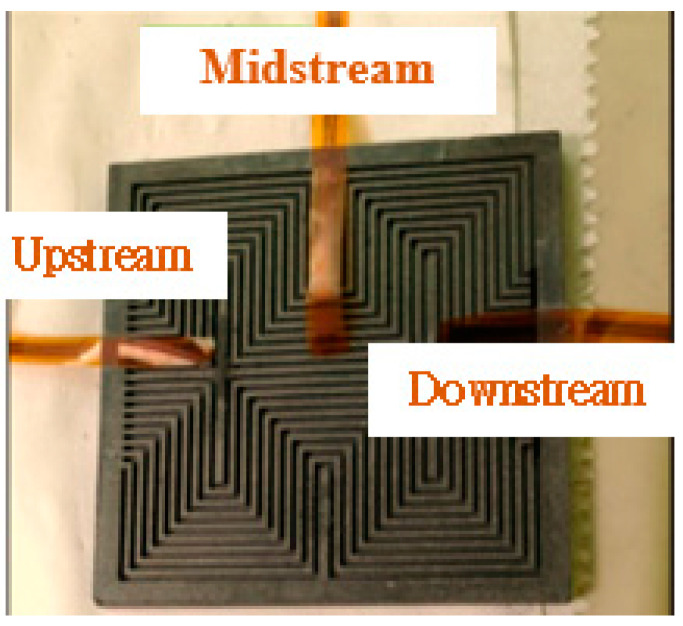
Flexible integrated microsensor embedding position.

**Figure 10:**
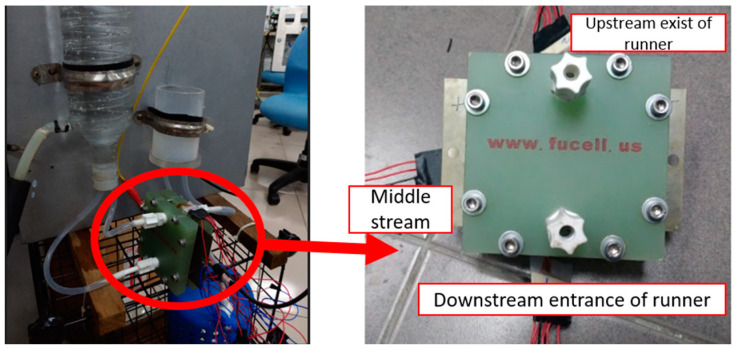
Physical image of flexible integrated microsensor embedded in the PEM water electrolyzer.

In order to observe the local physical change inside the PEM water electrolysis cell stack, the flexible integrated microsensor is embedded above the runner plate of PEM water electrolysis cell stack without influencing the performance of the PEM water electrolysis cell stack (Figure 9 and Figure 10). Finally, the PEM water electrolysis cell stack is closed uniformly by the closing pressure of the end plate, so that the signals of the flexible integrated microsensor can be captured stably. The flexible integrated microsensor is embedded in the PEM water electrolysis cell stack for a 100-h accelerated aging test, so as to understand the real-time microscopic distribution of important physical quantities from activation to aging of the PEM water electrolysis cell stack.

### 3.1. 100-h Accelerated Aging Test Conditions for PEM Water Electrolysis Cell Stack

**Figure 11:**
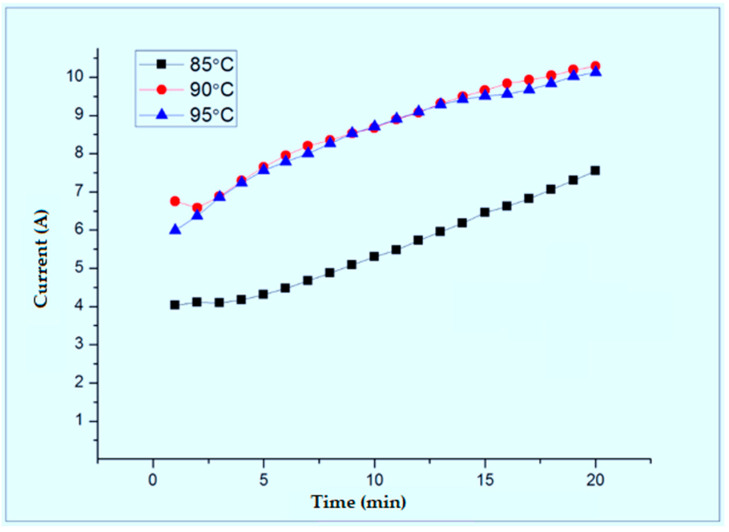
Performance curves of the PEM water electrolysis cell stack at different operating temperatures.

The performance of the PEM water electrolysis cell stack is tested at different temperatures (85 °C, 90 °C and 95 °C), as shown in Figure 11. It is found that the performance is enhanced as the temperature rises, but the performance declines at the back end after 95 °C, so 95 °C and a high voltage of 3 V are selected as 100-h accelerated aging test conditions, because a voltage of more than 3V will damage the catalyst.

### 3.2. Local Temperature and Voltage in 100-h Accelerated Aging of PEM Water Electrolysis Cell Stack 

**Figure 12:**
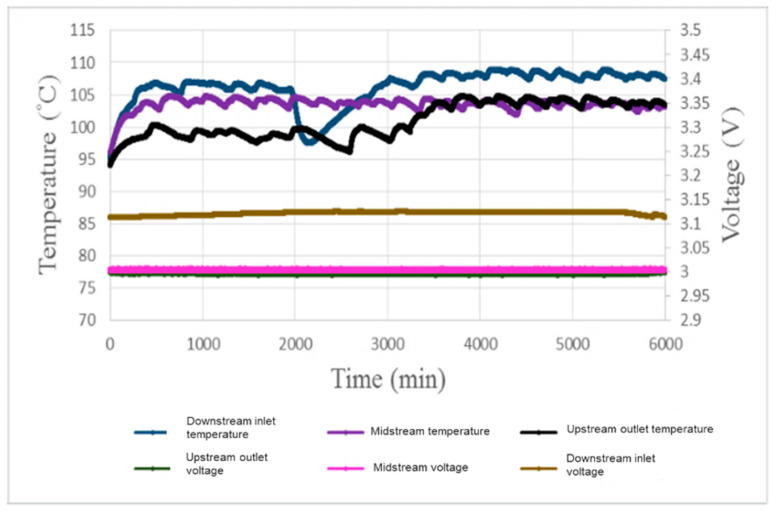
Local temperature and voltage in 100-h accelerated aging of the PEM water electrolysis cell stack.

Figure 12 shows the local temperature and voltage test for 100-h accelerated aging of the PEM water electrolysis cell stack. It is found that the temperature rises gradually with the operating time. The temperature at the downstream inlet is apparently higher than other regions. The temperature begins to fall at about 3 h; this may because mass inflow of fluid at the beginning of the reaction results in intense reaction, and a lot of gas is generated, inducing hot stack, and the fluid reduces the heat over time, so the temperature drops. The midstream temperature fluctuation is slighter than the other regions, meaning that the runner design is relatively free of the hot stack problem. The upstream is nearer to the outlet and close to air which dissipates heat, so the temperature is, relatively, the lowest. This result shows that the local heat distribution inside the PEM water electrolysis cell stack is more severe and fluctuates largely at the downstream inlet. The voltage distribution is steadier than the temperature distribution, and the temperature is higher only at the downstream inlet; this may be because the fluid is sufficient, inducing higher voltage. The midstream and upstream have lower voltage, which may be the result of the ohmic resistance and charge transfer resistance of the anode [8].

### 3.3. Local Current Density in 100-h Accelerated Aging of PEM Water Electrolysis Cell Stack

**Figure 13:**
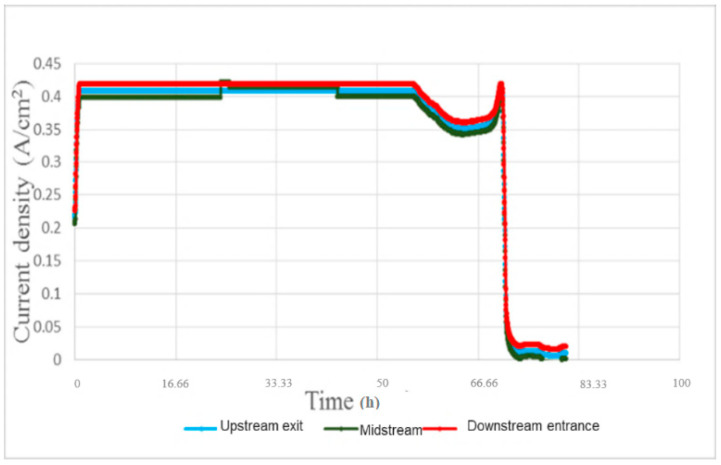
Local current density in 100-h accelerated aging of the PEM water electrolysis cell stack.

**Figure 14:**
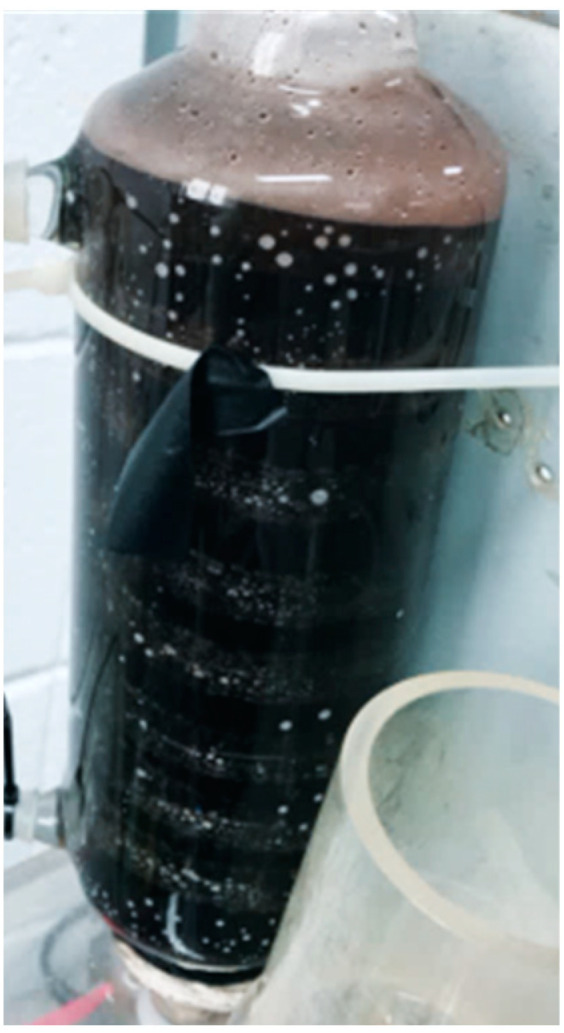
External Deionized water (DI) water glass tube.

Figure 13 shows the local current density test for 100-h accelerated aging of the PEM water electrolysis cell stack. It is observed that the downstream outlet has a slightly higher current density than the midstream and upstream outlets. The performance of the PEM water electrolysis cell stack reaches its maximum limit about 0.408184 A/cm^2^ after about 40 min, and then the reaction is stabilized. The performance begins to decline at about 55 h, meaning the MEA (Membrane Electrode Assembly) and runner plate of PEM water electrolysis cell stack have aged gradually, the external glass tube with Deionized water (DI) water has been blackened, and the dissolved graphite has been discharged from the outlet, as shown in Figure 14.

### 3.4. Local Flow in 100-h Accelerated Aging of PEM Water Electrolysis Cell Stack

**Figure 15:**
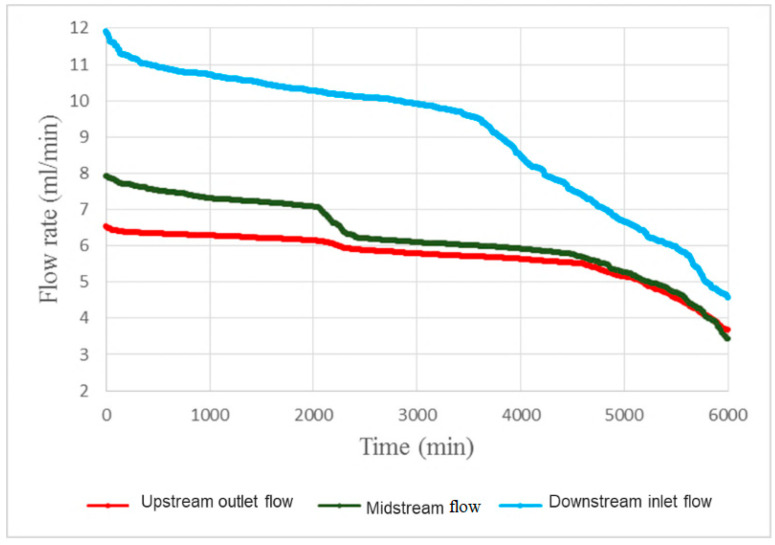
Local flow in 100-h accelerated aging of the PEM water electrolysis cell stack.

**Figure 16:**
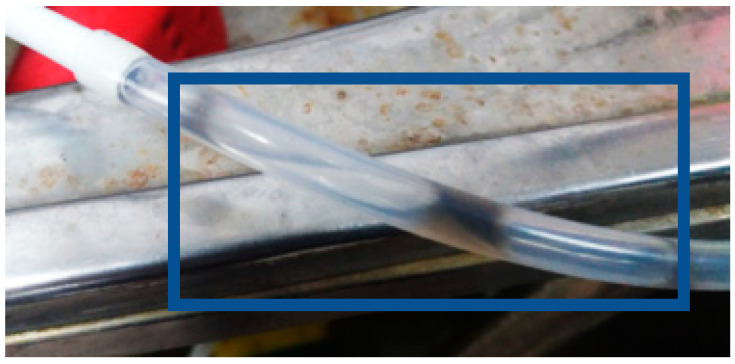
PEM water electrolysis cell stack anode outlet.

Figure 15 shows the local flow test for 100-h accelerated aging of the PEM water electrolysis cell stack. It is observed that the downstream outlet has the maximum flow, the runners maintain stable flow before 40 h, and the midstream runner flow begins to decrease rapidly after 40 h. Thus, it can be seen that the blocking of the reaction between the downstream and midstream may be a symptom of aging, and the aging becomes increasingly severe with time. The deposition of graphite plate and carbon paper results in deceleration of the runner blocking flow. The PEM water electrolysis cell stack anode outlet diagram in Figure 16 shows that the black graphite blocks the runner.

### 3.5. Analysis of Internal Parts of PEM Water Electrolysis Cell Stack after Accelerated Aging Test

**Figure 17:**
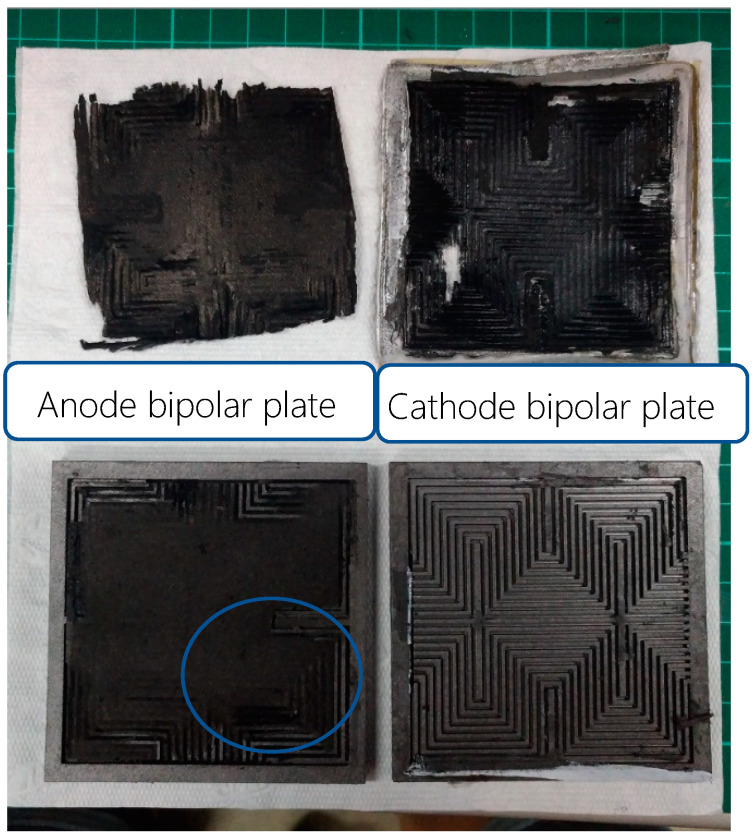
Internal parts of the PEM water electrolysis cell stack.

**Figure 18:**
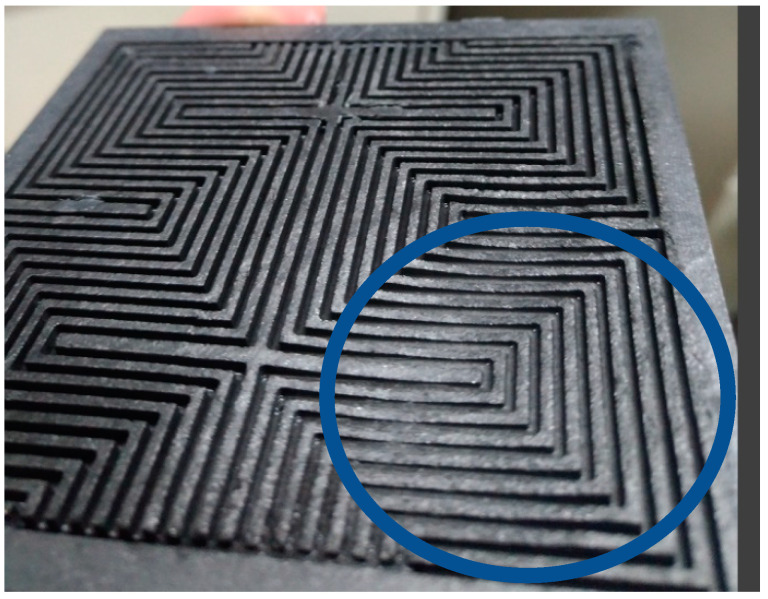
Close-up view of middle and lower reaches of the anode runner.

After the 100-h accelerated aging test for the PEM water electrolysis cell stack, the PEM water electrolysis cell stack is disassembled to ascertain the internal condition, as shown in Figure 17 and Figure 18. It is obvious that the regions of the runner plate close to the midstream and downstream are corroded, meaning the reaction is intense.

## 4. Conclusions

This study has successfully developed a flexible integrated microsensor by using MEMS technology. This flexible integrated microsensor has five sensing functions, it is resistant to high-temperature electrochemical environment, it can perform real-time measurement, and it can be placed in any position inside the PEM water electrolysis cell stack.

The flexible integrated microsensor was successfully embedded in the PEM water electrolysis cell stack, and the local important information inside the PEM water electrolysis cell stack was extracted successfully without influencing the operation. A 100-h accelerated aging test was performed. The test result showed that the key factors influencing the PEM water electrolysis cell stack were temperature and voltage. As the PEM water electrolysis cell stack aged internally, the nonuniform distribution of temperature and flow resulted in large temperature difference, lower current density, graphitization of the internal runner plate and carbon paper pyrolysis. The most severe condition occurred between the downstream inlet and the midstream.
